# New Insights on Quality, Safety, Nutritional, and Nutraceutical Properties of Honeydew Honeys from Italy

**DOI:** 10.3390/molecules30020410

**Published:** 2025-01-19

**Authors:** Andrea Mara, Federica Mainente, Vasiliki Soursou, Yolanda Picó, Iratxe Perales, Asma Ghorab, Gavino Sanna, Isabel Borrás-Linares, Gianni Zoccatelli, Marco Ciulu

**Affiliations:** 1 Department of Chemical, Physical, Mathematical and Natural Sciences, University of Sassari, Via Vienna 2, 07100 Sassari, Italy; amara@uniss.it (A.M.); sanna@uniss.it (G.S.); 2 Department of Biotechnology, University of Verona, Strada le Grazie 15, 37134 Verona, Italy; federica.mainente@univr.it (F.M.); gianni.zoccatelli@univr.it (G.Z.); 3 Environmental & Food Safety Research Group of the University of Valencia (SAMA-UV), Desertification Research Centre CIDE (CSIC-UV-GV), Road CV-315 Km 10.7, 46113 Moncada, Spain; vasiliki.soursou@uv.es (V.S.); yolanda.pico@uv.es (Y.P.); 4 Microfy Systems SL, Avda. Carrilet 243, 1-2, 08907 Barcelona, Spain; iratxe.perales@microfy.ai (I.P.); asma.ghorab@microfy.ai (A.G.); 5 Department of Vegetal Biology and Soil Sciences, Facultade de Ciencias, Universidade de Vigo, 32004 Ourense, Spain; 6 Department of Analytical Chemistry, Faculty of Sciences, University of Granada, Avda Fuentenueva s/n, 18071 Granada, Spain; iborras@ugr.es

**Keywords:** honeydew honey, minerals, phenolics, pesticide residues, antioxidant capacity, HMF, electrical conductivity, color

## Abstract

Honeydew honey is less studied than nectar honey, although it is characterized by peculiar nutritional properties. This is mainly due to its challenging production, which leads to easy counterfeiting and difficult valorization. This contribution aims to provide a comprehensive characterization of the physico-chemical, palynological, functional, and food safety properties of a large sampling of honeydew honeys collected throughout Italy. The honeydew elements, conductivity, color, antioxidant properties, total polyphenol content, hydroxymethylfurfural, major and trace elements, toxic and rare earth elements, and pesticide residues were measured in 59 samples of honeydew honey from forest, eucalyptus, fir, oak, and citrus sources. Physico-chemical and antioxidant properties were unable to differentiate the botanical origin of Italian honeydew honeys. Similarly, the mineral composition did not vary significantly, whereas rare earth elements appeared to be promising markers for classifying their origin. Multivariate analysis allowed discriminating fir honeydews from the other varieties. Concerning safety aspects, pesticide residues were detected in 90% of the samples, with fir honeydews exhibiting the lowest contamination levels, probably due to its production in less industrialized areas. Acetamiprid and imidacloprid were the most prevalent pesticide residues, but their concentrations were below the limit indicated by the EFSA. These findings suggest the need for a continuous monitoring program for contaminants to ensure safety and to assess risk.

## 1. Introduction

Honey is a sweet food produced by bees (*Apis mellifera*) that is recognized for its unique nutraceutical and therapeutic properties [1]. These features make honey a precious food with significant economic value, and therefore, it is often subjected to fraudulent practices, such as adulteration or mislabeling [2]. Recent surveys conducted by the European Commission have highlighted the need for new strategies aimed at safeguarding and valorizing beehive products [3]. Honey can be produced by foraging bees collecting nectar from flowers or honeydew from plants. Honeydew is derived from the secretions of plants or the excretions of plant-sucking insects such as aphids or mealybugs. This synergic action makes honeydew honey a peculiar product with distinct properties to blossom honey [4]. In comparison, honeydew honeys are typically characterized by a higher electrical conductivity, pH, acidity, and ash content [5].

Similarly, they are normally darker in color and characterized by a higher content of minerals [6] and antioxidant compounds, such as polyphenols [7]. The saccharide composition of honeydew honeys is usually more complex than blossom honeys, and specific sugars can be employed as markers to distinguish honeydew honeys from blossom honeys or to identify their botanical origin [8]. For example, trisaccharides such as raffinose, erlose and, mainly, melizitose are proposed as chemical markers for discriminating between honeydew and blossom honeys [9].

From a commercial perspective, blossom honeys are typically preferred due to their organoleptic properties. This is because honeydew honeys are generally characterized by an intense aromatic flavor and a bitter taste [10]. Nevertheless, their antioxidant and antibacterial properties appeal to consumers seeking beneficial products [11]. In comparison to blossom ones, honeydew honeys are less frequently available on the market. The production of honeydew honey is influenced by the use of agrochemicals in crops, which has resulted in a decline in the population of honeydew-producing insects [12,13]. Similarly, producing honeydew honey is more challenging because the limited resources also prompt bees to search for nectar [14]. Consequently, honeys produced in geographical areas where both sources are available are not easily classifiable as honeydew honey or blossom honey [8]. Therefore, many efforts may be conducted to safeguard and valorize honeydew honey production [3].

The valorization of honeydew honeys involves characterizing all aspects that differentiate these honeys from blossom honeys, such as the content of antioxidant substances or minerals [15]. On the other hand, the safeguarding of these honeys depends on developing new analytical methods to distinguish between blossom and honeydew honeys [16,17] and determine the botanical source of the honeydew [8,18]. These aspects can be evaluated using mass spectrometry-based methodologies [19] for the determination of saccharide composition [20,21], amino acids and proteins [22,23], polyphenolic compounds [24,25], and minerals or elements [26,27,28,29] that can be used as botanical or geographical markers [30]. In this context, spectroscopic methodologies also have great applications for botanical and geographical classification [31,32,33,34], the prediction of chemical–physical parameters [35,36], and the detection of adulterants [37,38], also using portable instrumentation to trace origin and detect adulterants simultaneously [39].

Despite several studies in this field, few have examined a statistically significant number of honeydew samples from different botanical origins, as revealed by Seraglio et al. [11] in a recent review. The most studied variety of honeydew honey is the Bracatinga honey, typical of South America [40,41]. Other commonly investigated varieties are honeydew honeys from pine [14,42], fir [42,43], and oak [44,45], while citrus, hazelnut, and eucalyptus honeys are typical of Mediterranean areas [8,46]. Conversely, in numerous studies, the botanical provenance of honeydew is either unidentified or unknown [11]. Considering the lack of robustness of most published data and the high number of factors that may influence the composition of honey, it is evident that more comprehensive studies are necessary, based on a representative sampling that considers both botanical and geographical origin.

Concerning Italian honeys, Persano Oddo et al. [47] measured the physicochemical characteristics and the saccharide profile of 14 unifloral honeys. Among others, fir honeydew honey and honey from *Metcalfa pruinosa* honeydew were analyzed. Regarding the inorganic fraction, the elemental composition of the honeys was utilized either independently [48] or in conjunction with the measurement of stable isotope ratios [49] for the classification of Italian nectar and honeydew honey according to their botanical origin. Moreover, the quantification of ionic species enabled the differentiation between Italian and Western Balkan samples and the distinction between nectar and honeydew honeys [50]. In addition, the content of phenolic compounds and antioxidant activities [51,52,53], the saccharide composition [21], and the volatile profile [54,55] have been shown to be useful for honey authentication purposes.

Despite the significant contribution of these studies, the sampling of honeydew honeys was often critical, or the samples were categorized without considering the different botanical origins of the honeydew. Moreover, the food safety aspects related to the content of pesticide residues have not been investigated.

This study aims to characterize Italian honeydew honey from diverse botanical sources in terms of food safety and parameters that normally differentiate honeydew honey from blossom honey. The collection included samples from almost all Italian regions and a variety of botanical sources, including forest, eucalyptus, fir, oak, citrus, and hazelnut.

The ratio of honeydew elements (HDEs) to the total pollen grain content was determined to ascertain whether each sample met the traditional criteria for classification as “honeydew honey”. Subsequently, two physicochemical properties, namely electrical conductivity and color, which have the potential to differentiate honeydew honey from blossom honey, were evaluated. The hydroxymethylfurfural (HMF) content was analyzed to assess the freshness of the samples, and their antioxidant properties were evaluated in terms of total polyphenol content (TPC) and, primarily, through both 2,2-diphenyl-1-picrylhydrazyl (DPPH) and 2,2-diphenyl-1-picrylhydrazyl (ABTS) assays.

Moreover, this study quantified the concentrations of nutritionally relevant elements along with lanthanides, which could prove useful in the georeferencing of samples. In conclusion, the levels of both food safety and environmental contamination of the honeydew honeys, as well as of their collection sites, were evaluated through the analysis of toxic elements and pesticide residues.

## 2. Results

### 2.1. Honeydew Elements, Antioxidant Properties, Conductivity, and Color

An analysis of HDEs has been conducted to assess the correct origin attribution and declaration. High antioxidant properties generally characterize honeydew honey; therefore, they have been determined using both DPPH and ABTS assays, as the antioxidant activity may vary depending on the test employed [51]. In addition, the total polyphenol content has been determined, as these compounds are characterized by higher antioxidant activity [56]. HMF has been measured, as it is correlated to honey freshness and can be related to antioxidant properties [57]. Finally, conductivity and color have been characterized, as they usually differentiate honeydew honey from nectar honey [7].

**Figure 1:**
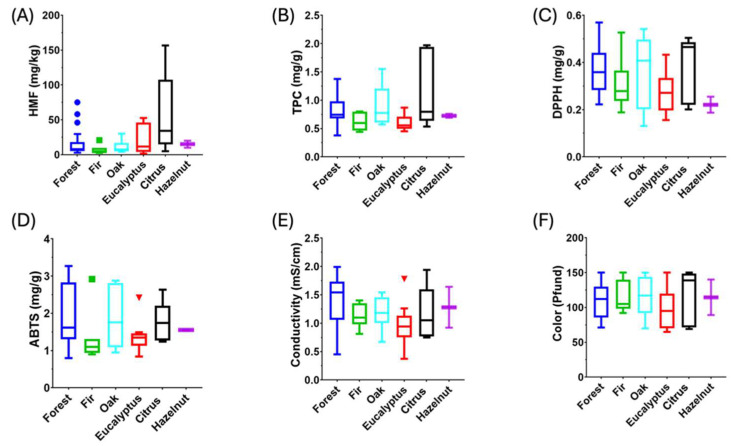
(**A**) Hydroxymethylfurfural, (**B**) total polyphenol content, (**C**) radical scavenging activity, (**D**) ABTS assay, (**E**) conductivity, and (**F**) color of honeydew honeys.

The HDE ratio (Table S1) ranged from 0.03 to values higher than 3, with an average value of 0.56. Honey can be declared “honeydew honey” when the HDE value exceeds three and the conductivity is above 0.8 mS cm^−1^ [58]. In this study, a few samples exceeded the HDE threshold. Previous studies of honeydew honeys produced in Spain have reported similar results, including analyses of oak honeys from Galicia (northwestern Spain), honeydew honeys from Tenerife, or pine honeys from Greece [9,59,60,61,62]. These results are commonly reported in the literature and occur when pollen-rich samples are analyzed. In these cases, the HDE ratio is not useful for classification. Considering the acquired pollen spectra, conductivity, and measured color (Figure 1), all the samples were classified as honeydew honey.

Figure 1 shows the results obtained for the analysis of conductivity and color, antioxidant properties, HMF, and total polyphenol content. No significative differences (*p* < 0.05) were observed for each parameter between varying honeydew botanical origins. The content of HMF was generally lower than the limit of 40 mg kg^−1^ set by the European Union [63], indicating acceptable freshness of the samples. Two samples from eucalyptus and one from citrus honeydew exceeded the limit.

The samples were collected in 2022 and 2023. Therefore, the results can also be compared as a function of the year of production. However, no significant differences have been observed. Table S2 reports the average value (±standard deviation) and range for each parameter with varying botanical honeydew origins.

### 2.2. Minerals, Toxic Elements, and Rare Earth Elements

**Figure 2:**
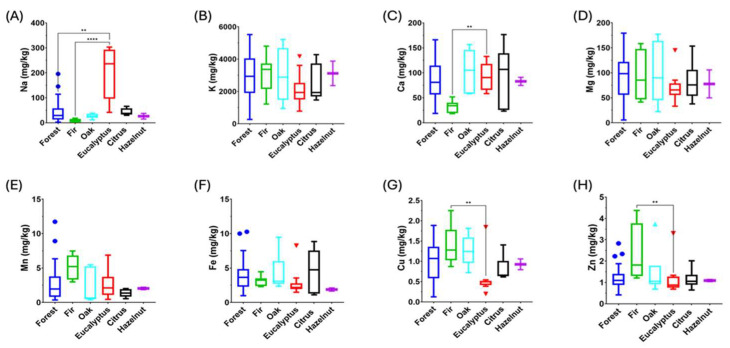
Minerals in Italian honeydew honeys. (**A**) Sodium, (**B**) potassium, (**C**) calcium, (**D**) magnesium, (**E**) manganese, (**F**) iron, (**G**) copper, and (**H**) zinc. (**) *p* < 0.01, (****) *p* < 0.0001.

Elements in honey are related to botanical and geographical origins [28] and contribute to their nutritional and antioxidant properties [64]. They also reflect anthropogenic activities, and the presence of potentially toxic elements may be related to environmental pollution [65]. Moreover, ultra-trace elements such as rare earth elements can be used as geographical markers [26,28]. Figure 2 displays the concentration of minerals (elements of nutritional interest) in honeydew honeys, whereas Table S2 reports the concentration of each element as a function of the honeydew botanical origin.

As reported in Figure 2, minerals may vary according to botanical origin. Statistical differences have been observed for some elements except for K, Mg, Mn, and Fe (*p* < 0.05). In general, the most abundant element is K, followed by Na, Mg, and Ca, depending on the samples, whereas concentrations of Mn and Fe are higher than those of Cu and Zn. In decreasing order of concentration, other abundant elements are Rb and Al, whereas Ni, Sr, and Ba are present at trace levels. Potentially toxic elements such as As, Cd, Hg, Pb, Sn, and Tl are often below the relevant limit of quantification [27,28], while U is often quantified but at concentrations almost always below the μg/kg level. It can therefore be concluded that the content of potentially toxic elements is of no toxicological interest.

**Figure 3:**
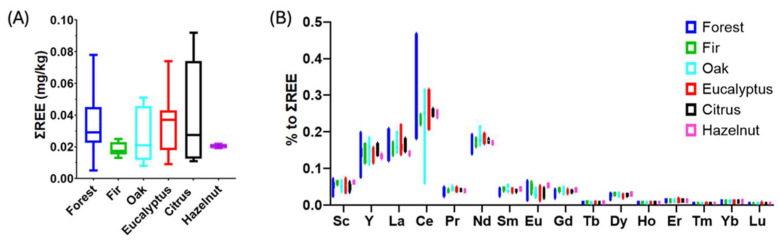
Rare earth elements in Italian honeydew honeys: (**A**) total REE amount and (**B**) relative amount of single REEs.

Finally, rare earth elements (REEs) are found in ultra-trace concentrations. Table S2 reports average values and ranges, while Figure 3 compares the results obtained in terms of the total REE content and the percentage of each element as a function of the botanical origin. No significant differences are observed between the different categories (*p* < 0.05), although the fir and hazelnut samples exhibit a lower average REE content. In terms of percent composition, the most abundant element is Ce and, in general, the abundance reflects the one predicted by the Oddo–Harkins rule [28]. The only exception is for Eu, which is, in some cases, higher than Sm and Gd. The data show that the abundance of light REEs is more variable than heavy REEs.

### 2.3. Pesticides Residues

The samples were analyzed with methods from the literature [66,67] developed for the quantification of pesticides in bee products, including the most relevant miticides used by beekeepers against varroa parasites, as well as many insecticides, fungicides, herbicides, and nematocides extensively used in crop protection. Table 1 shows the pesticides detected at least once, and the data are listed according to botanical origin.

### 2.4. Principal Component Analysis

**Figure 4:**
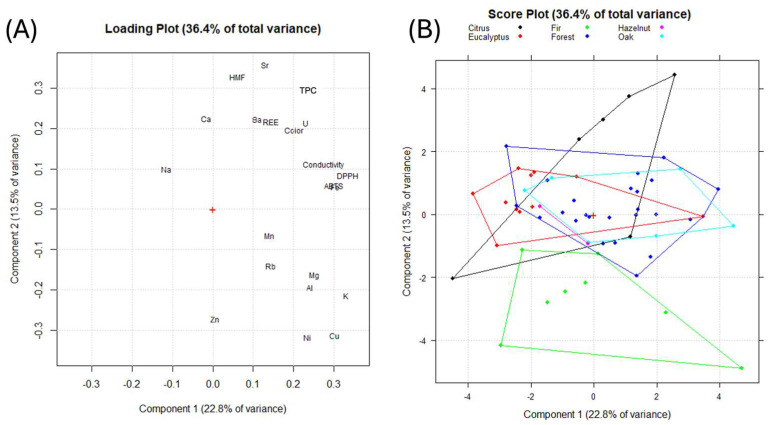
Principal component analysis of the parameters analyzed in Italian honeydew honeys. Objects are colored according to their botanical origin. (**A**) Loading plot and (**B**) score plot.

Principal component analysis (PCA) was performed using data describing the antioxidant properties, TPC, HMF, conductivity, color, minerals, and REEs, excluding pesticides, as they are rarely quantified. Similarly, potentially toxic elements were also excluded from the dataset. Due to the high correlation between REEs, they were considered in terms of total content. Figure 4 reports the results we obtained.

From the loading plot (Figure 4A), positive PC1 values indicate a higher content of abundant elements (K, Cu, Fe), antioxidant activities, and conductivity. PC2 discriminates the content of Zn, Ni, and Cu by negative values and the content of Sr, Ba, REEs, HMF, and Ca by positive values. As expected, DPPH and ABTS are highly correlated with each other, and both are highly correlated with conductivity and Fe. In particular, HMF is correlated with alkali-earth elements such as Sr, Ca, and Ba, and the latter is correlated with REEs and U. Looking at the score plot (Figure 4B), samples are colored according to the botanical origin, and groups are centered to the origin, except for fir samples, which are characterized by negative PC2 values. This is the only category that is well separated from the others, as fir samples are characterized by higher amounts of Zn and lower concentrations of REEs, TPC, and HMF.

## 3. Discussion

In this work, the content of total polyphenols, HMF, conductivity, color, antioxidant properties, minerals, potentially toxic elements, and pesticide residues in forest, fir, oak, eucalyptus, citrus, and hazelnut honeydew honeys produced in Italy were characterized.

Except for a few parameters, the analyzed samples generally showed no significant differences concerning botanical honeydew origin. The samples of fir honeydew honey showed lower contents of HMF and REEs and higher contents of Zn and Cu. Another class of samples that differed from the others was that of eucalyptus samples, which had a significantly higher Na content. These samples come mainly from Sardinia island. In this case, the observed difference could be attributed to a specific geographic and botanical origin.

As reported in previous studies [4], antioxidant properties and minerals discriminate well between blossom and honeydew honeys. However, when comparing honeydew honeys of different botanical origins, the variability of these parameters does not allow any discrimination. In this case, the saccharide profile or other minor components such as amino acids or proteins may help in the discrimination of botanical origin [8].

The obtained results were compared with those that were previously published in similar studies. The HMF content of the samples is close to that previously measured in other Italian samples [68]. Also in comparison with other countries, our samples showed values similar to those measured in oak samples from Spain [69] or Bracatinga honeydew honeys [70]. Concerning antioxidant properties, our results can be compared with those of Castiglioni et al. [51] who employed DPPH and ABTS assays to analyze Italian honeydew honeys, also determining the total polyphenol content. Their results are in fair agreement with those reported in this study, with the TPC of fir samples being similar, whereas the values of antioxidant activities were found to be lower in this study. Our conductivity results are in the range previously reported for Italian samples [11,47], but these values are higher than those measured by Nešović et al. [5] in samples from Montenegro.

The content of elements can be compared with several worldwide studies that have used these descriptors for both botanical and geographic origin classification [71]. However, only a few of them involved honeydew honey samples from Italy. Bontempo et al. [49] measured the stable isotope ratios and elemental composition of Italian honeydew honeys. Considering the high composition variability and the concentration ranges, the data from the literature are in good agreement with those obtained in this study, with the sole exception of the Na content. As previously stated, in this study, eucalyptus honeydew honey samples showed a higher content of Na than other botanical origins. Similarly, in the study of Bontempo et al. [49], eucalyptus blossom honeys exhibited higher values of Na (authors did not declare the region of provenance), and this also was revealed in our previous work about the elemental composition of blossom honeys from Sardinia and Spain [28]. Therefore, assuming that most of the eucalyptus honeys on the Italian market come from Sardinia [72], the elevated sodium content may be a distinctive characteristic of Sardinian eucalyptus honeys derived from both nectar or honeydew.

The content of REEs can be compared with previous data reported for Italian blossom honeys [28,73], whereas, to the best of our knowledge, this is the first study that measured REEs in Italian honeydew honeys. In comparison with Italian multifloral honeys [28,73], the honeydew samples of this study have a higher average REE content. However, when compared to unifloral honeys of strawberry tree and thistle, the REE content is found to be similar [28]. Although no statistical differences (*p* < 0.05) were observed between the total amount of REEs in eucalyptus nectar honeys and eucalyptus honeydew honeys [28], the distribution of these elements is quite different. Honeydew samples have a lower percentage of light REEs (La–Nd) and a higher percentage of heavy REEs (Gd–Lu). Therefore, REE fingerprints may also be effective in differentiating nectar and honeydew honeys from the same botanical sources. Finally, the REE fingerprints can be also compared with data reported for Bracatinga honeydew honey from Brazil [41]. The comparison highlights significant differences that suggest possible geographical discrimination using these descriptors.

Multivariate analysis of the aforementioned parameters was performed mainly for visualization purposes and to check for correlation between parameters. Although the construction of a classification model was not possible, the PCA showed that fir honeydews differed from the other varieties, mainly based on Zn, Ni, and Cu contents. This observation is significant as it allows us to understand, in general terms, which honeydew variety is the most peculiar from a chemical–physical point of view, and more specifically, which of the parameters taken into consideration can be the subject of further investigation in the perspective of authenticating fir honeydews. From the point of view of the information that can be obtained from the adopted chemometric approach, even though the explained variance is not particularly high (<50%), it must be remembered that this finding is common in other multivariate analysis studies where the elemental profile is taken into consideration [27,28].

Concerning the content of pesticide residues, it should first be noted that only six out of fifty-nine (approximately 10%) analyzed honeys were free of contaminants. These data confirm the ability of honey, and in this case honeydew honey, to reflect the ‘state of health’ of the territory by revealing important information on the use of products that are potentially harmful to humans, the environment, and pollinating insects. While it is true that the presence of residues in honey samples is highly dependent on the agricultural practices in use around the hive, it is important to understand how the origin and the floral source of honeydew can be revelatory of such practices, and for this reason, it was decided to report the concentration of residues by each declared botanical origin. In this context, it is evident that fir honeydew tends to be free of such residues, constituting a product with a lower risk for the consumer. This observation may depend on the very nature of fir honeydews, which are normally produced in mountainous areas, far from large urban agglomerations, or areas used for intensive cultivation and therefore less subject to the use of pesticides.

On the contrary, eucalyptus honeydews, produced mainly on the island of Sardinia, show a wider variety of residues being produced in an area with a strong agricultural vocation and where, therefore, pesticide use is more prominent.

Going into the specifics of the detected and quantified residues, acetamiprid is the most frequent contaminant. This product, which falls into the category of neonicotinoids, was introduced in the past as an alternative to carbamates and organophosphates and is used as an insecticide in a variety of crops. Its presence in honey is certainly of interest due to the growing body of scientific evidence demonstrating the toxicity of acetamiprid in various mammalian organs [74]. The measured concentrations are in line with those found in other studies [75] and are below the maximum residual limits indicated in the latest EFSA opinion [76]. However, these limits do not consider any potential synergistic effects that may be exerted by residues that are simultaneously present in honey. Therefore, the observed frequent detection of acetamiprid should also merit special attention. As expected, the highest levels of acetamiprid are found in citrus honeydew, due to its extensive use in citrus cultivation. Among neonicotinoids, imidacloprid is the second most traced contaminant in honeydew samples. Like acetamiprid, it is a versatile product used for different types of crops and represents a potential threat to pollinating insects [77]. The levels of imidacloprid are also below those suggested by the EFSA, but considerations on potential synergism always apply. Although scientific studies show that honeydew is a possible source of exposure to neonicotinoid insecticides for pollinating insects, to the best of our knowledge, no data have been found on the presence of imidacloprid in honeydew honeys. The presence of atrazine, in its form as it is or in its degradation products (e.g., deisopropyl atrazine), is certainly of interest. This herbicide was banned in Europe in 2004 but continues to be traced in environmental matrices even today [78]. Honeydews have already shown their ability to accumulate this contaminant in the past. For example, atrazine has been quantified in the honeydew of *Mimosa scabrella* from Brazil [79].

In conclusion, it is important to address the presence of residues associated with veterinary drugs utilized in beekeeping practices. Coumaphos, a phosphorothioate used in the control of Varroa, has always been found largely below the maximum residue level (MRL) suggested by the EFSA for honey (0.01 mg/kg, [77]). Conversely, quite high concentrations of 2,4-dimethylphenyl-N′-methylformamidine (DMPF), an environmental degradation product of the pesticide amitraz, largely used by beekeepers against Varroa, have been found in eucalyptus honeydews. In one instance, an estimated concentration of 90 ng/g has been observed, which is close to the MRL limits (i.e., 0.2 mg/kg) suggested by the EFSA [77]. It is important to remember that for coumaphos and amitraz, there are specific maximum residual limits, given by Commission Regulation (EU) No. 37/2010 of 22 December 2009 on pharmacologically active substances and their classification regarding maximum residue limits in foodstuffs of animal origin. More specifically, MRLs of 100 and 200 µg/kg have been established for coumaphos and amitraz, respectively.

## 4. Materials and Methods

### 4.1. Sample Collection

**Figure 5:**
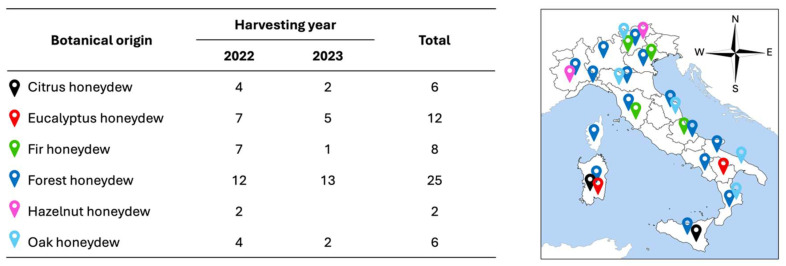
Italian honeydew honey sampling, key information, and geographical provenance.

A total of 59 honeydew honeys from Italy with different botanical origins were collected between 2022 and 2023. The varieties we investigated were forest (n = 25), eucalyptus (n = 12), fir (n = 8), oak (n = 6), citrus (n = 6), and hazelnut (n = 2) honeydew honey (Table S1). The botanical sources reflect the flora of the country, whereas the sampling size reflects the sample availability from local or online markets and beekeepers. Botanical origin was assessed by pollen analysis. Figure 5 summarizes the key information about the dataset and the geographical provenance of the samples. The samples were stored in the dark at 4 °C until analysis.

### 4.2. Honey AI Analysis

Samples were analyzed using Honey.AI, a pioneer automated microscope specifically designed for the honey industry, which works with image processing and deep learning models to perform rapid analysis of key honey quality parameters such as color, pollen spectrum, HDE, and conductivity. The automated device has been developed by Microfy Systems (Barcelona, Spain) [80] and integrates a light brightfield microscope with x10/x40 objectives and a 3MP digital camera. After sample homogenization, 6 g of honey was weighed and diluted with distilled water to 25 mL and then analyzed for conductivity using the probe integrated into the microscope following standard procedures. Color was measured in PFund using a 4X objective and specific disposable plastic cuvettes. The color analysis was developed by establishing an AI correlation between PFund, measured with a Hanna Instrument, and RGB color grading, measured with OpenCV and image processing algorithms. The microscope accurately correlates RGB color with light transmittance (max. ± 4 error). As for HDE analysis, 10 g of honey was dissolved in 20 mL of tap water. The solution was then centrifuged at 4000× *g* for 10 min. The supernatant was discarded, and the sediment was resuspended with 25 µL of glycerinated water, collected, and placed on a 24 × 24 slide. The analysis of honeydew elements was conducted automatically using a 40X objective (for some dirty honeys, 0.5% acidulated water was used). The HDE ratio was calculated based on the identification and quantification of various fungal elements (e.g., Cladosporium, Spores, Metschnikowia yeast), other spores produced by plant pathogens (e.g., Leptosphaeria, Stemphylium, Alternaria, Urediniospores, Pleospora, Basidiospores, etc.), as well as hyphae and microalgae. The HDE ratio was obtained by dividing the number of honeydew elements over the total number of pollen grains counted.

### 4.3. Analysis of Total Polyphenols

The total polyphenol content (TPC) was determined by the Folin–Ciocalteu method, as described by Castiglioni et al. [51]. In a 96-well microplate, 120 μL of Folin–Ciocalteu reagent (previously diluted at 1:10, *v*/*v*, with water) was added to 5 μL of a 4% (*w*/*v*) honey solution and incubated for 10 min in the dark. Finally, 80 μL of 10% (*w*/*v*) sodium carbonate solution was added. The microplate was shaken and left to stand for 2 h in the dark. The absorbance was measured at a wavelength of 760 nm against water as the blank with a Tecan Infinite 200 Pro microplate reader (Tecan Trading AG, Mannedorf, Switzerland). Gallic acid (GA) in the 1.56 (265.4 mg L^−1^) – 0.05 mM (8.5 mg L^−1^) concentration range was used as a standard. The results were expressed as mg gallic acid equivalents (mg GAE) per g honey.

### 4.4. Analysis of Antioxidant Properties

The antioxidant activity of honey samples was evaluated using the DPPH assay [51] with some modifications. In a 96-well microplate, 80 μL of a 5% (*w*/*v*) honey solution was mixed with 160 μL of a 0.2 mM ethanolic DPPH solution. After 15 min of incubation at room temperature in the dark, the absorbance of the solution at a wavelength of 517 nm was read against water as the blank. Trolox (TE) in the 0.15 (37.5 mg L^−1^)–0.005 mM 1.25 mg L^−1^) concentration range was used as a standard, and the results were expressed as mg Trolox equivalents (mg TE) per g honey.

The antioxidant activity of honey samples was also assessed using the ABTS assay, as described by De Marchi et al. [81]. The stock solution of ABTS·+ was prepared by mixing a 7.4 mM methanolic ABTS solution with a 2.46 mM aqueous solution of K_2_S_2_O_8_ in a 1:1 (*v*/*v*) ratio and allowed to stand for 12 h at room temperature in the dark. A freshly prepared working solution was diluted with methanol to reach an absorbance of 0.9 ± 0.1 at 734 nm. For the assay, 20 μL of a (*w*/*v*) honey solution was placed in microplate wells, and 200 μL of the working solution was added. The decrease in absorbance was monitored at 734 nm using a Tecan Infinite 200 Pro microplate reader (Tecan Trading AG, Mannedorf, Switzerland). Water was used as the blank, and Trolox (TE) in the 0.3–0.005 mM concentration range was used as a standard. The results were expressed as mg Trolox equivalents (mg TE) per g honey.

### 4.5. Analysis of Hydroxymethylfurfural

Hydroxymethylfurfural (HMF) content was determined by reverse-phase chromatography, as described by Vorlová et al. [82]. The method, originally developed for other saccharide-rich foodstuffs, was optimized for the honey matrix and validated in terms of sensitivity, linear dynamic range, and precision. All the validation parameters were satisfactory in relation to the analytical purposes. Honey samples (5% *w*/*v*) were analyzed using an Extrema LC-4000 system (Jasco Europe, Verona, Italy) equipped with a Eurospher II 100-5 C18 column (i.d. 4.6 × 250 mm, 5 μm particle size, Knauer Wissenschaftliche Geräte GmbH, Berlin, Germany). The mobile phase was 10% methanol HPLC grade with a 1 mL/min flow rate. The sample injection volume was 20 μL and the column temperature was 25 °C. A nine-point calibration curve of HMF (H9877 purity ≥ 99%, Sigma-Aldrich, Steinheim, Germany), whose absorbance was measured at a wavelength of 285 nm (R^2^ = 0.999), was used to evaluate HMF content in honey.

### 4.6. Elemental Analysis

This method for the elemental analysis in honey samples has been previously developed and validated [27,28]. Samples were prepared by using microwave digestion for the analysis of major elements, trace elements, and toxic elements, whereas dry ashing was employed for lanthanide analysis. Microwave acid digestion was conducted using an ultraWAVE SRC from Milestone (Sorisole, Italy). Thus, approximately 0.700 g of honey was weighed in 15 mL PTFE vessels and treated with 0.5 mL of HNO_3_, 3 mL of H_2_O_2_, and 4 mL of type I water. After digestion at 240 °C, the samples were collected, diluted to 15 mL, and filtered before analysis. Dry ashing was performed using a Controller P320 muffle from Nabertherm (Lilienthal, Germany) and weighing about 5.0 g of honey in porcelain crucibles of 150 mL. After ashing at 600 °C, the samples were treated with 10 mL of 5% HNO_3_ aqueous solution, diluted to 15 mL, and filtered before analysis. Elemental analysis was performed using a NexION 300X ICP-MS spectrometer from Perkin Elmer (Milan, Italy) for the determination of macro elements (i.e., Na, Mg, K, Ca), trace and toxic elements (i.e., Al, As, Ba, Cd, Cu, Fe, Hg, Mn, Ni, Pb, Sn, Rb, Sr, U, Tl, Zn), and rare earth elements (i.e., Sc, Y, La, Ce, Pr, Nd, Sm, Eu, Gd, Dy, Ho, Er, Tm, Yb, Lu). For further information about the instrumental settings and validation parameters, please see references [27,28].

### 4.7. Analysis of Pesticides

Analyte extraction was performed using the QuEChERS approach that was previously developed and validated by Calatayud-Vernich et al. [66]. Briefly, 5 g of honey was weighed into 50 mL centrifuge tubes and then added to 7.5 mL of water, 10 mL of acetonitrile, 6 g of MgSO_4_, and 1 g of NaCl. After homogenization, the mixture was centrifuged for 5 min at 3500× *g*. Then, 2 mL of the supernatant was transferred to a 15 mL centrifuge tube, and 50 mg C18, 50 mg PSA, and 150 mg MgSO_4_ were added. After a second homogenization, the mix was vortexed and centrifuged for 5 min at 3500× *g*. Finally, the supernatant was filtered using a 13 mm × 0.22 mm PTFE into autosampler vials for analysis.

Sample analysis was performed by ultra-high-pressure liquid chromatography coupled with high-resolution mass spectrometry (UHPLC-HRMS/MS). Liquid chromatography was achieved on a Vanquish system (Thermo Fisher Scientific, Whaltham MA, USA). The conditions for pesticide analysis were the following: analytical column Luna^®^ C18 150 × 2 mm (3 µm particle size, Phenomenex, Torrance, CA, USA), column temperature of 30 °C, injection volume 5 µL, mobile phase water (A) and methanol (B), both with 5 mM of ammonium formate, and a flow rate of 0.3 mL/min. A linear gradient was performed as follows: 0 min (50% B), 10 min (83% B), 12 min (83% B), 12.5 min (98% B), 15 min (98% B), 16 min (50% B), and finally an equilibration time of 10 min. Mass spectrometry (MS) analysis was performed on an Orbitrap Exploris 120 mass spectrometer (Thermo Fisher Scientific, Whaltham MA, USA) equipped with a heated electrospray ionization probe (HESI). Raw pesticide data were acquired using Tracefinder software (v.4.0, Thermo Fisher Scientific, Whaltham MA, USA).

Pesticide standards were purchased in their solid form (purity > 99%) from Merck (Darmstadt, Germany) and solutions of 1000 mg L^−1^ were prepared using methanol obtained from Avantor (Radnor PA, USA). These solutions were used to create a mix of pesticides that was employed for the calibration curves. Calibration curves were constructed using eight concentration levels, namely 5, 10, 25, 50, 75, 100, 250, and 500 ng mL^−1^. The complete list of pesticides analyzed for this study is reported in Table S3.

### 4.8. Statistical Analysis

Data analysis was conducted using GraphPad Prism (v. 9.1.0 221) and Chemometric Agile Tool (CAT) [83]. The Shapiro–Wilk test was used to check the normal distribution. Considering the group size and the results of the Shapiro–Wilk test, the Kruskal–Wallis test was employed for multiple comparisons, and Dunn’s test was used as a post hoc test. PCA was performed for data visualization. Statistical significance was set at *p* < 0.05.

## 5. Conclusions

This comprehensive study provides novel insights into the quality, safety, nutritional, and nutraceutical properties of Italian honeydew honeys derived from diverse botanical origins. Antioxidant properties, total polyphenol content, hydroxymethylfurfural levels, conductivity, color, and minerals did not exhibit significant variations among samples from different botanical origins. The samples studied here displayed physicochemical properties analogous to those observed in other European honeydew honeys while exhibiting distinctive elemental profiles. Concentrations of toxic elements were below levels of concern. Rare earth element profiles demonstrated potential for geographical origin discrimination, with light REEs exhibiting greater variability compared to heavy REEs. Pesticide residues were detected in 90% of the samples, at concentrations always below the relevant MRLs. The most prevalent residues were found to be acetamiprid and imidacloprid. Fir honeydew honey exhibited the lowest pesticide concentrations, as it is mainly produced in areas with low agricultural pressure.

In conclusion, the data reported here support the high nutritional value of these honeys while emphasizing the necessity for continued monitoring of pesticide residues. Further investigations should be conducted to use honeydew honeys as pollution indicators of environmental health status.

## Figures and Tables

**Table 1:** Pesticide residues detected in Italian honeydew honeys.

	Forest (n = 25)		Fir (n = 8)		Oak (n = 6)		Eucalyptus (n = 12)		Citrus (n = 6)		Hazelnut (n = 2)	
	Mean ± sd	(Min–Median–Max)	Mean ± sd	(Min–Median–Max)	Mean ± sd	(Min–Median–Max)	Mean ± sd	(Min–Median–Max)	Mean ± sd	(Min–Median–Max)	Mean ± sd	(Min–Median–Max)
Acetamiprid	2 ± 2	(0.1–1–8)	2 ± 3	(0.3–2–4)	4 ± 6	(0.2–0–11)	0.3 ± 0.3	(0.04–0.3–0.7)	5 ± 4	(1–4–10)	0.1 ± 0.1	(0.04–0.1–0.2)
Atrazine	nd		nd		nd		nd		nd		0.125 ± 0.006	(0.125–0.125–0.125)
Atrazine-deisopropyl	7 ± 9	(2–2–17)	nd		1 ± 1	(0.1–0–2)	nd		nd		nd	
Azoxyxtrobin	nd		nd		nd		22 ± 1	(22–22–22)	nd		nd	
Bensulfuron methyl	nd		nd		nd		17.4 ± 0.9	(17.4–17.4–17.4)	nd		nd	
Bentazone	16.8 ± 0.8	(16.8–16.8–16.8)	nd		nd		nd		nd		nd	
Carbendazime	7 ± 1	(6-7-7)	nd		nd		nd		nd		0.58 ± 0.03	(0.58–0.58–0.58)
Carbofuran-3-hydroxy	13 ± 6	(0.4–15–16)	nd		nd		7 ± 6	(0.2–11–12)	nd		nd	
Chlorfenvinphos	nd		nd		nd		nd		2 ± 2	(0.1–1–4)	0.24 ± 0.01	(0.24–0.24–0.24)
Coumaphos	0.4 ± 0.3	(0.1–0.3–1.1)	0.3 ± 0.02	(0.3–0.3–0.3)	4.6 ± 0.2	(4.6–4.6–4.6)	1.09 ± 0.05	(1.04–1.09–1.14)	nd		±	
Cyhalothrin	nd		nd		nd		41 ± 2	(41–41–41)	nd		±	
Difenoconzole	nd		nd		nd		15.5 ± 0.8	(15.5–15.5–15.5)	nd		±	
DMPF	3 ± 2	(1–3–5)	nd		nd		40 ± 50	(0.1–20–90)	5 ± 6	(1–5–10)	±	
Fluvalinate	nd		nd		nd		36 ± 2	(36–36–36)	nd		±	
Imazamox	nd		nd		nd		19 ± 1	(19–19–19)	nd		±	
Imidacloprid	2 ± 2	(0.3–1–7)	0.2 ± 0.1	(0.1–0.2–0.3)	nd		nd		0.8 ± 0.5	(0.3–0.7–1.5)	0.19 ± 0.04	(0.16–0.19–0.21)
Omethoate	nd		nd		nd		20 ± 1	(20–20–20)	nd		nd	
Propazine	nd		nd		nd		0.092 ± 0.005	(0.092–0.092–0.092)	nd		0.127 ± 0.006	(0.127–0.127–0.127)
Pyriproxifen	1.59 ± 0.08	(1.59–1.59–1.59)	nd		nd		nd		nd		2.8 ± 0.1	(2.8–2.8–2.8)
Tebuconazole	1.29 ± 0.06	(1.29–1.29–1.29)	nd		nd		nd		nd		nd	
Terbuthylazine	1.11 ± 0.06	(1.11–1.11–1.11)	nd		nd		nd		1.16 ± 0.06	(1.16–1.16–1.16)	nd	
Tricyclazol	nd		nd		nd		22 ± 1	(22–22–22)	nd		nd	

All data are expressed as ng/g. nd = not detected.

## Data Availability

All data are available in Supplementary Materials. Further inquiries can be directed to the corresponding author.

## Supplementary Materials

The following supporting information can be downloaded at: https://www.mdpi.com/article/10.3390/molecules30020410/s1, Table S1: Dataset, botanical origins, harvesting year, provenance and HDE ratio for each honeydew honey sample from Italy; Table S2: Antioxidant properties, minerals, and rare earth elements in Italian honeydew honeys. Mean ± standard deviation and range; Table S3: List of the pesticides analyzed.

## References

[1] Bogdanov S., Jurendic T., Sieber R., Gallmann P. (2008). Honey for Nutrition and Health: A Review. J. Am. Coll. Nutr..

[2] Thrasyvoulou A., Tananaki C., Goras G., Karazafiris E., Dimou M., Liolios V., Kanelis D., Gounari S. (2018). Legislation of Honey Criteria and Standards. J. Apic. Res..

[3] European Commission EU Coordinated Action “From the Hives” (Honey 2021–2022). https://food.ec.europa.eu/safety/eu-agri-food-fraud-network/eu-coordinated-actions/honey-2021-2022_en#qapdf.

[4] Pita-Calvo C., Vázquez M. (2017). Differences between Honeydew and Blossom Honeys: A Review. Trends Food Sci. Technol..

[5] Nešović M., Gašić U., Tosti T., Trifković J., Baošić R., Blagojević S., Ignjatović L., Tešić Ž. (2020). Physicochemical Analysis and Phenolic Profile of Polyfloral and Honeydew Honey from Montenegro. RSC Adv..

[6] Solayman M., Islam M.A., Paul S., Ali Y., Khalil M.I., Alam N., Gan S.H. (2016). Physicochemical Properties, Minerals, Trace Elements, and Heavy Metals in Honey of Different Origins: A Comprehensive Review. Comp. Rev. Food Sci. Food Safe.

[7] Recklies K., Peukert C., Kölling-Speer I., Speer K. (2021). Differentiation of Honeydew Honeys from Blossom Honeys and According to Their Botanical Origin by Electrical Conductivity and Phenolic and Sugar Spectra. J. Agric. Food Chem..

[8] Pita-Calvo C., Vázquez M. (2018). Honeydew Honeys: A Review on the Characterization and Authentication of Botanical and Geographical Origins. J. Agric. Food Chem..

[9] Bentabol Manzanares A., García Z.H., Galdón B.R., Rodríguez E.R., Romero C.D. (2011). Differentiation of Blossom and Honeydew Honeys Using Multivariate Analysis on the Physicochemical Parameters and Sugar Composition. Food Chem..

[10] Machado A.M., Miguel M.G., Vilas-Boas M., Figueiredo A.C. (2020). Honey Volatiles as a Fingerprint for Botanical Origin—A Review on Their Occurrence on Monofloral Honeys. Molecules.

[11] Seraglio S.K.T., Silva B., Bergamo G., Brugnerotto P., Gonzaga L.V., Fett R., Costa A.C.O. (2019). An Overview of Physicochemical Characteristics and Health-Promoting Properties of Honeydew Honey. Food Res. Int..

[12] Alma A., Ferracini C., Burgio G. (2005). Development of a Sequential Plan to Evaluate *Neodryinus typhlocybae* (Ashmead) (Hymenoptera: Dryinidae) Population Associated with *Metcalfa pruinosa* (Say) (Homoptera: Flatidae) Infestation in Northwestern Italy. Env. Entomol..

[13] Balakhnina I.V., Pastarnak I.N., Gnezdilov V.M. (2014). Monitoring and Control of Metcalfa Pruinosa (Say) (Hemiptera, Auchenorrhyncha: Flatidae) in Krasnodar Territory. Entmol. Rev..

[14] Gounari S., Zotos C.E., Dafnis S.D., Moschidis G., Papadopoulos G.K. (2023). On the Impact of Critical Factors to Honeydew Honey Production: The Case of *Marchalina hellenica* and Pine Honey. J. Apic. Res..

[15] Tarapatskyy M., Sowa P., Zaguła G., Dżugan M., Puchalski C. (2021). Assessment of the Botanical Origin of Polish Honeys Based on Physicochemical Properties and Bioactive Components with Chemometric Analysis. Molecules.

[16] Bergamo G., Seraglio S.K.T., Gonzaga L.V., Fett R., Costa A.C.O. (2019). Physicochemical Characteristics of Bracatinga Honeydew Honey and Blossom Honey Produced in the State of Santa Catarina: An Approach to Honey Differentiation. Food Res. Int..

[17] García-Seval V., Martínez-Alfaro C., Saurina J., Núñez O., Sentellas S. (2022). Characterization, Classification and Authentication of Spanish Blossom and Honeydew Honeys by Non-Targeted HPLC-UV and Off-Line SPE HPLC-UV Polyphenolic Fingerprinting Strategies. Foods.

[18] Kaškonienė V., Venskutonis P.R. (2010). Floral Markers in Honey of Various Botanical and Geographic Origins: A Review. Comp. Rev. Food Sci. Food Safe.

[19] Quirantes-Piné R., Sanna G., Mara A., Borrás-Linares I., Mainente F., Picó Y., Zoccatelli G., Lozano-Sánchez J., Ciulu M. (2024). Mass Spectrometry Characterization of Honeydew Honey: A Critical Review. Foods.

[20] Blaško J., Fulín M., Kubinec R., DUHAČKOVÁ Ľ., Kubincová J., Kukurová K., Blažková M., Kunštek M., GÁBRIŠOVA Ľ.I., Kafková V. (2023). Fast Differentiation of Floral and Honeydew Honeys Using Gas Chromatography-Mass Spectrometry. J. Food Nutr. Res..

[21] Tedesco R., Barbaro E., Zangrando R., Rizzoli A., Malagnini V., Gambaro A., Fontana P., Capodaglio G. (2020). Carbohydrate Determination in Honey Samples by Ion Chromatography–Mass Spectrometry (HPAEC-MS). Anal. Bioanal. Chem..

[22] Erban T., Shcherbachenko E., Talacko P., Harant K. (2021). A Single Honey Proteome Dataset for Identifying Adulteration by Foreign Amylases and Mining Various Protein Markers Natural to Honey. J. Proteom..

[23] Łozowicka B., Kaczyński P., Iwaniuk P. (2021). Analysis of 22 Free Amino Acids in Honey from Eastern Europe and Central Asia Using LC-MS/MS Technique without Derivatization Step. J. Food Compos. Anal..

[24] Seraglio S.K.T., Valese A.C., Daguer H., Bergamo G., Azevedo M.S., Gonzaga L.V., Fett R., Costa A.C.O. (2016). Development and Validation of a LC-ESI-MS/MS Method for the Determination of Phenolic Compounds in Honeydew Honeys with the Diluted-and-Shoot Approach. Food Res. Int..

[25] García-Seval V., Saurina J., Sentellas S., Núñez O. (2022). Off-Line SPE LC-LRMS Polyphenolic Fingerprinting and Chemometrics to Classify and Authenticate Spanish Honey. Molecules.

[26] Drivelos S.A., Danezis G.P., Halagarda M., Popek S., Georgiou C.A. (2021). Geographical Origin and Botanical Type Honey Authentication through Elemental Metabolomics via Chemometrics. Food Chem..

[27] Mara A., Deidda S., Caredda M., Ciulu M., Deroma M., Farinini E., Floris I., Langasco I., Leardi R., Pilo M.I. (2022). Multi-Elemental Analysis as a Tool to Ascertain the Safety and the Origin of Beehive Products: Development, Validation, and Application of an ICP-MS Method on Four Unifloral Honeys Produced in Sardinia, Italy. Molecules.

[28] Mara A., Migliorini M., Ciulu M., Chignola R., Egido C., Núñez O., Sentellas S., Saurina J., Caredda M., Deroma M.A. (2024). Elemental Fingerprinting Combined with Machine Learning Techniques as a Powerful Tool for Geographical Discrimination of Honeys from Nearby Regions. Foods.

[29] Magdas D.A., Guyon F., Puscas R., Vigouroux A., Gaillard L., Dehelean A., Feher I., Cristea G. (2021). Applications of Emerging Stable Isotopes and Elemental Markers for Geographical and Varietal Recognition of Romanian and French Honeys. Food Chem..

[30] Tsagkaris A.S., Koulis G.A., Danezis G.P., Martakos I., Dasenaki M., Georgiou C.A., Thomaidis N.S. (2021). Honey Authenticity: Analytical Techniques, State of the Art and Challenges. RSC Adv..

[31] Ciulu M., Oertel E., Serra R., Farre R., Spano N., Caredda M., Malfatti L., Sanna G. (2020). Classification of Unifloral Honeys from SARDINIA (Italy) by ATR-FTIR Spectroscopy and Random Forest. Molecules.

[32] Caredda M., Mara A., Ciulu M., Floris I., Pilo M.I., Spano N., Sanna G. (2023). Use of Genetic Algorithms in the Wavelength Selection of FT-MIR Spectra to Classify Unifloral Honeys from Sardinia. Food Control.

[33] David M., Magdas D.A. (2024). Authentication of Honey Origin and Harvesting Year Based on Raman Spectroscopy and Chemometrics. Talanta Open.

[34] David M., Hategan A.R., Berghian-Grosan C., Magdas D.A. (2022). The Development of Honey Recognition Models Based on the Association between ATR-IR Spectroscopy and Advanced Statistical Tools. IJMS.

[35] Escuredo O., Rodríguez-Flores M.S., Meno L., Seijo M.C. (2021). Prediction of Physicochemical Properties in Honeys with Portable Near-Infrared (microNIR) Spectroscopy Combined with Multivariate Data Processing. Foods.

[36] Pauliuc D., Ciursă P., Ropciuc S., Dranca F., Oroian M. (2021). Physicochemical Parameters Prediction and Authentication of Different Monofloral Honeys Based on FTIR Spectra. J. Food Compos. Anal..

[37] Valinger D., Longin L., Grbeš F., Benković M., Jurina T., Gajdoš Kljusurić J., Jurinjak Tušek A. (2021). Detection of Honey Adulteration—The Potential of UV-VIS and NIR Spectroscopy Coupled with Multivariate Analysis. LWT.

[38] Cárdenas-Escudero J., Galán-Madruga D., Cáceres J.O. (2023). Rapid, Reliable and Easy-to-Perform Chemometric-Less Method for Rice Syrup Adulterated Honey Detection Using FTIR-ATR. Talanta.

[39] Caredda M., Ciulu M., Tilocca F., Langasco I., Núñez O., Sentellas S., Saurina J., Pilo M.I., Spano N., Sanna G. (2024). Portable NIR Spectroscopy to Simultaneously Trace Honey Botanical and Geographical Origins and Detect Syrup Adulteration. Foods.

[40] Azevedo M.S., Seraglio S.K.T., Rocha G., Balderas C.B., Piovezan M., Gonzaga L.V., Falkenberg D.D.B., Fett R., De Oliveira M.A.L., Costa A.C.O. (2017). Free Amino Acid Determination by GC-MS Combined with a Chemometric Approach for Geographical Classification of Bracatinga Honeydew Honey (Mimosa Scabrella Bentham). Food Control.

[41] Silva B., Gonzaga L.V., Maltez H.F., Samochvalov K.B., Fett R., Costa A.C.O. (2021). Elemental Profiling by ICP-MS as a Tool for Geographical Discrimination: The Case of Bracatinga Honeydew Honey. J. Food Compos. Anal..

[42] Karabagias I.K., Vlasiou M., Kontakos S., Drouza C., Kontominas M.G., Keramidas A.D. (2018). Geographical Discrimination of Pine and Fir Honeys Using Multivariate Analyses of Major and Minor Honey Components Identified by 1H NMR and HPLC along with Physicochemical Data. Eur. Food Res. Technol..

[43] Lušić D., Koprivnjak O., Ćurić D., Sabatini A. (2007). Volatile Profile of Croatian Lime Tree (*Tilia* sp.), Fir Honeydew (*Abies alba*) and Sage (*Salvia officinalis*) Honey. Food Technol. Biotechnol..

[44] Hernanz D., Jara-Palacios M.J., Santos J.L., Gómez Pajuelo A., Heredia F.J., Terrab A. (2023). The Profile of Phenolic Compounds by HPLC-MS in Spanish Oak (Quercus) Honeydew Honey and Their Relationships with Color and Antioxidant Activity. LWT.

[45] Jerković I., Marijanović Z. (2010). Oak (Quercus Frainetto Ten.) Honeydew Honey—Approach to Screening of Volatile Organic Composition and Antioxidant Capacity (DPPH and FRAP Assay). Molecules.

[46] Karabagias I.K., Louppis A.P., Karabournioti S., Kontakos S., Papastephanou C., Kontominas M.G. (2017). Characterization and Geographical Discrimination of Commercial Citrus Spp. Honeys Produced in Different Mediterranean Countries Based on Minerals, Volatile Compounds and Physicochemical Parameters, Using Chemometrics. Food Chem..

[47] Persano Oddo L., Piazza M.G., Sabatini A.G., Accorti M. (1995). Characterization of Unifloral Honeys. Apidologie.

[48] Conti M.E., Stripeikis J., Campanella L., Cucina D., Tudino M.B. (2007). Characterization of Italian Honeys (Marche Region) on the Basis of Their Mineral Content and Some Typical Quality Parameters. Chem. Cent. J..

[49] Bontempo L., Camin F., Ziller L., Perini M., Nicolini G., Larcher R. (2017). Isotopic and Elemental Composition of Selected Types of Italian Honey. Measurement.

[50] Fermo P., Beretta G., Maffei Facino R., Gelmini F., Piazzalunga A. (2013). Ionic Profile of Honey as a Potential Indicator of Botanical Origin and Global Environmental Pollution. Environ. Pollut..

[51] Castiglioni S., Stefano M., Astolfi P., Carloni P. (2017). Chemometric Approach to the Analysis of Antioxidant Properties and Colour of Typical Italian Monofloral Honeys. Int. J. Food Sci. Tech..

[52] Di Marco G., Gismondi A., Panzanella L., Canuti L., Impei S., Leonardi D., Canini A. (2018). Botanical Influence on Phenolic Profile and Antioxidant Level of Italian Honeys. J. Food Sci. Technol..

[53] Preti R., Tarola A.M. (2022). Chemometric Evaluation of the Antioxidant Properties and Phenolic Compounds in Italian Honeys as Markers of Floral Origin. Eur. Food Res. Technol..

[54] Tedesco R., Scalabrin E., Malagnini V., Strojnik L., Ogrinc N., Capodaglio G. (2022). Characterization of Botanical Origin of Italian Honey by Carbohydrate Composition and Volatile Organic Compounds (VOCs). Foods.

[55] Breschi C., Ieri F., Calamai L., Miele A., D’Agostino S., Melani F., Zanoni B., Mulinacci N., Cecchi L. (2024). HS-SPME-GC-MS Analysis of the Volatile Composition of Italian Honey for Its Characterization and Authentication Using the Genetic Algorithm. Separations.

[56] Martinello M., Mutinelli F. (2021). Antioxidant Activity in Bee Products: A Review. Antioxidants.

[57] Salis S., Spano N., Ciulu M., Floris I., Pilo M.I., Sanna G. (2021). Electrochemical Determination of the “Furanic Index” in Honey. Molecules.

[58] Louveaux J., Maurizio A., Vorwohl G. (1978). Methods of Melissopalynology. Bee World.

[59] Carmen Seijo M., Escuredo O., Fernández-González M. (2011). Fungal Diversity in Honeys from Northwest Spain and Their Relationship to the Ecological Origin of the Product. Grana.

[60] Olga E., María F.-G., Carmen S.M. (2012). Differentiation of Blossom Honey and Honeydew Honey from Northwest Spain. Agriculture.

[61] Rodríguez-Flores M.S., Escuredo O., Míguez M., Seijo M.C. (2019). Differentiation of Oak Honeydew and Chestnut Honeys from the Same Geographical Origin Using Chemometric Methods. Food Chem..

[62] Dimou M., Katsaros J., Klonari K.T., Thrasyvoulou A. (2006). Discriminating Pine and Fir Honeydew Honeys by Microscopic Characteristics. J. Apic. Res..

[63] European Community COUNCIL DIRECTIVE 2001/110/EC of 20 December 2001 Relating to Honey. https://eur-lex.europa.eu/eli/dir/2001/110/oj/eng.

[64] Bodó A., Radványi L., Kőszegi T., Csepregi R., Nagy D.U., Farkas Á., Kocsis M. (2021). Quality Evaluation of Light- and Dark-Colored Hungarian Honeys, Focusing on Botanical Origin, Antioxidant Capacity and Mineral Content. Molecules.

[65] Bargańska Ż., Ślebioda M., Namieśnik J. (2016). Honey Bees and Their Products: Bioindicators of Environmental Contamination. Crit. Rev. Environ. Sci. Technol..

[66] Calatayud-Vernich P., Calatayud F., Simó E., Picó Y. (2016). Efficiency of QuEChERS Approach for Determining 52 Pesticide Residues in Honey and Honey Bees. MethodsX.

[67] Calatayud-Vernich P., Calatayud F., Simó E., Picó Y. (2018). Pesticide Residues in Honey Bees, Pollen and Beeswax: Assessing Beehive Exposure. Environ. Pollut..

[68] Apriceno A., Girelli A.M., Scuto F.R., Tarola A.M. (2018). Determination of Furanic Compounds and Acidity for Italian Honey Quality. Flavour. Fragr. J..

[69] Shantal Rodríguez Flores M., Escuredo O., Carmen Seijo M. (2015). Assessment of Physicochemical and Antioxidant Characteristics of Quercus Pyrenaica Honeydew Honeys. Food Chem..

[70] Silva B., Brugnerotto P., Seraglio S.K.T., Bergamo G., Biluca F.C., Santos A.C.D., Braghini F., Schulz M., Colombo C.H., Samochvalov K.B. (2022). Physicochemical, Phenolic, and Mineral Characterization of Mimosa Scabrella Bentham Honeydew Honey: A Trial for Obtaining the Geographical Identification. J. Food Compos. Anal..

[71] Pohl P., Bielawska-Pohl A., Dzimitrowicz A., Jamroz P., Welna M., Lesniewicz A., Szymczycha-Madeja A. (2017). Recent Achievements in Element Analysis of Bee Honeys by Atomic and Mass Spectrometry Methods. TrAC Trends Anal. Chem..

[72] Floris I., Satta A., Ruiu L. (2007). Honeys of Sardinia (Italy). J. Apic. Res..

[73] Squadrone S., Brizio P., Stella C., Pederiva S., Brusa F., Mogliotti P., Garrone A., Abete M.C. (2020). Trace and Rare Earth Elements in Monofloral and Multifloral Honeys from Northwestern Italy; A First Attempt of Characterization by a Multi-Elemental Profile. J. Trace Elem. Med. Biol..

[74] Phogat A., Singh J., Kumar V., Malik V. (2022). Toxicity of the Acetamiprid Insecticide for Mammals: A Review. Env. Chem. Lett..

[75] Kędzierska-Matysek M., Teter A., Skałecki P., Topyła B., Domaradzki P., Poleszak E., Florek M. (2022). Residues of Pesticides and Heavy Metals in Polish Varietal Honey. Foods.

[76] Bellisai G., Bernasconi G., Brancato A., Cabrera L.C., Castellan I., Ferreira L., Giner G., Greco L., Jarrah S., EFSA (European Food Safety Authority) (2022). Modification of the Existing Maximum Residue Levels for Acetamiprid in Honey and Various Oilseed Crops. EFS2.

[77] European Food Safety Authority (EFSA) (2016). Setting of Maximum Residue Levels for Amitraz, Coumaphos, Flumequine, Oxytetracycline, Permethrin and Streptomycin in Certain Products of Animal Origin. EFS2.

[78] Triassi M., Montuori P., Provvisiero D.P., De Rosa E., Di Duca F., Sarnacchiaro P., Díez S. (2022). Occurrence and Spatial-Temporal Distribution of Atrazine and Its Metabolites in the Aquatic Environment of the Volturno River Estuary, Southern Italy. Sci. Total Environ..

[79] Brugnerotto P., Costa A.C.O., Fuente-Ballesteros A., Ares A.M., Gonzaga L.V., Fett R., Bernal J. (2023). Determination of Seven Pesticide Residues in Mimosa Scabrella Honeydew Honey from Brazil by GC-MS. J. Food Compos. Anal..

[80] Microfy.AI. Honey.AI Microscope. https://microfy.ai/en/our-technology/honey-ai.

[81] De Marchi L., Salemi L., Bellumori M., Chignola R., Mainente F., Santisteban Soto D.V., Fierri I., Ciulu M., Zoccatelli G. (2024). Thermal Degradation of Red Cabbage (*Brassica oleracea* L. Var. Capitata f. Rubra) Anthocyanins in a Water Model Extract under Accelerated Shelf-Life Testing. Food Chem..

[82] Kalábová L., Večerek V. (2006). Hydroxymethylfurfural Contents in Foodstuffs Determined by HPLC Method. J. Food Nutr. Res..

[83] Leardi R., Melzi C., Polotti G. CAT (Chemometric Agile Tool). http://gruppochemiometria.it/index.php/software.

