# Supplementary material for: New Insights on Quality, Safety, Nutritional, and Nutraceutical Properties of Honeydew Honeys from Italy
Source: Molecules. 2025 Jan 19;30(2):410. doi: 10.3390/molecules30020410 (PMC11767624; doi:10.3390/molecules30020410)
Supplement: Supplementary file 1 [file molecules-30-00410-s001.zip › molecules-3405473-supplementary.pdf]

Table S1. Dataset, botanical origins, harvesting year, provenance and HDE ratio for each honeydew honey sample from Italy.

ID	Botanical origin	Harvesting year	Provenance	HDE ratio
2022MB1	Forest honeydew	2022	Italy and Spain	
2022MB2	Forest honeydew	2022	Italy	
2022MB4	Forest honeydew	2022	Piedmont	0,18
2022MB5	Forest honeydew	2022	Lombardy	> 3
2022MB6	Forest honeydew	2022	Veneto	0,08
2022MB7	Forest honeydew	2022	Veneto	0,47
2022MB8	Forest honeydew	2022	Lombardy	1,08
2022MB9	Forest honeydew	2022	Trentino-Alto Adige/Südtirol	> 3
2022MB10	Forest honeydew	2022	Tuscany	0,80
2022MB11	Forest honeydew	2022	Corsica	> 3
2022MB12	Forest honeydew	2022	Emilia-Romagna	0,15
2022MB13	Forest honeydew	2022	Italy	0,54
2023MB14	Forest honeydew	2023	Liguria	0,92
2023MB15	Forest honeydew	2023	Emilia Romagna	> 3
2023MB16	Forest honeydew	2023	Veneto	0,51
2023MB17	Forest honeydew	2023	Veneto	> 3
2023MB18	Forest honeydew	2023	Abruzzo	1,19
2023MB19	Forest honeydew	2023	San Marino	0,54
2023MB20	Forest honeydew	2023	Veneto	0,65
2023MB21	Forest honeydew	2023	Tuscany	0,28
2023MB23	Forest honeydew	2023	Lombardia	0,45
2023MB24	Forest honeydew	2023	Calabria	0,28
2023MB25	Forest honeydew	2023	Lombardia	> 3
2023MB26	Forest honeydew	2023	Campania	1,62
2023MB27	Forest honeydew	2023	Marche	> 3
2022MA1	Fir honeydew	2022	Trentino-Alto Adige/Südtirol	0,16
2022MA2	Fir honeydew	2022	Trentino-Alto Adige/Südtirol	1,14
2022MA3	Fir honeydew	2022	Veneto	0,03
2022MA4	Fir honeydew	2022	Veneto	0,14
2022MA5	Fir honeydew	2022	Tuscany	0,25
2022 MA6	Fir honeydew	2022	Abruzzo	0,91
2022MA7	Fir honeydew	2022	Abruzzo	0,69
2023MA8	Fir honeydew	2023	Tuscany	1,76

2022MQ1	Oak honeydew	2022	Trentino-Alto Adige/Südtirol	0,16
2022MQ2	Oak honeydew	2022	Marche	1,41
2022MQ3	Oak honeydew	2022	Calabria	1,06
2022MQ4	Oak honeydew	2022	Puglia	0,53
2023MQ5	Oak honeydew	2023	Puglia	1,43
2023MQ6	Oak honeydew	2023	Emilia Romagna	2,08
2022ME1	Eucalyptus honeydew	2022	Basilicata	0,08
2022ME2	Eucalyptus honeydew	2022	Basilicata	0,03
2022ME3	Eucalyptus honeydew	2022	Sardinia	0,25
2022ME4	Eucalyptus honeydew	2022	Sardinia	0,33
2022ME5	Eucalyptus honeydew	2022	Sardinia	0,19
2022ME6	Eucalyptus honeydew	2022	Sardinia	0,05
2022ME7	Eucalyptus honeydew	2022	Sardinia	0,93
2023ME8	Eucalyptus honeydew	2023	Sardinia	0,09
2023ME9	Eucalyptus honeydew	2023	Sardinia	0,13
2023ME10	Eucalyptus honeydew	2023	Sardinia	0,43
2023ME11	Eucalyptus honeydew	2023	Sardinia	0,90
2023ME12	Eucalyptus honeydew	2023	Sardinia	0,08
2022MAG1	Citrus honeydew	2022	Sicily	0,46
2022MAG2	Citrus honeydew	2022	Italy	0,46
2022MAG3	Citrus honeydew	2022	Sardinia	0,54
2022MAG4	Citrus honeydew	2022	Sardinia	
2023MAG5	Citrus honeydew	2023	Sicily	2,05
2023MAG6	Citrus honeydew	2023	Italy	1,14
2022MN1	Hazelnut honeydew	2022	Piedmont	1,72
2022MN2	Hazelnut honeydew	2022	Trentino-Alto Adige/Südtirol	0,20

HDE ratio expressed as ratio of honeydew elements over total pollen grain content

Table S2. Antioxidant properties, minerals, and rare earth elements in Italian honeydew honeys. Mean ± standard deviation and range.

	**Forest (n = 25)**		**Fir (n = 8)**		**Oak (n = 6)**		**Eucalyptus (n = 12)**		**Citrus (n = 6)**		**Hazelnut (n = 2)**	
	**mean ± sd**	**(min-median-max)**	**mean ± sd**	**(min-median-max)**	**mean ± sd**	**(min-median-max)**	**mean ± sd**	**(min-median-max)**	**mean ± sd**	**(min-median-max)**	**mean ± sd**	**(min-median-max)**
HMF (mg/kg)	20 ± 20	(3 - 10 - 80)	7 ± 7	(3 - 4 - 21)	10 ± 10	(4 - 10 - 30)	20 ± 20	(2 - 10 - 50)	60 ± 60	(5 - 30 - 160)	15 ± 7	(10 - 15 - 20)
TPP (mg/g)	0.8 ± 0.2	(0.4 - 0.7 - 1.4)	0.6 ± 0.1	(0.4 - 0.6 - 0.8)	0.9 ± 0.4	(0.6 - 0.8 - 1.5)	0.6 ± 0.1	(0.4 - 0.6 - 0.9)	1.2 ± 0.7	(0.5 - 0.8 - 2)	0.72 ± 0.05	(0.69 - 0.72 - 0.76)
DPPH (mg/g)	0.4 ± 0.1	(0.2 - 0.4 - 0.6)	0.3 ± 0.1	(0.2 - 0.3 - 0.5)	0.4 ± 0.2	(0.1 - 0.4 - 0.5)	0.27 ± 0.09	(0.16 - 0.27 - 0.43)	0.4 ± 0.1	(0.2 - 0.5 - 0.5)	0.22 ± 0.05	(0.19 - 0.22 - 0.25)
ABTS (mg/g)	2 ± 0.8	(0.8 - 1.6 - 3.3)	1.4 ± 0.7	(0.9 - 1.1 - 2.9)	1.9 ± 0.9	(1 - 1.8 - 2.9)	1.4 ± 0.4	(0.8 - 1.3 - 2.4)	1.7 ± 0.6	(1.2 - 1.7 - 2.6)	1.55 ± 0.01	(1.54 - 1.55 - 1.56)
Conductivity (mS/cm)	1.5 ± 0.3	(0.8 - 1.7 - 2)	1.1 ± 0.2	(0.8 - 1.1 - 1.4)	1.2 ± 0.3	(0.7 - 1.2 - 1.5)	1 ± 0.3	(0.4 - 0.9 - 1.8)	1.2 ± 0.5	(0.8 - 1.1 - 1.9)	1.3 ± 0.5	(0.9 - 1.3 - 1.6)
Color (Pfund)	110 ± 30	(70 - 110 - 150)	110 ± 20	(90 - 100 - 150)	120 ± 30	(70 - 120 - 150)	100 ± 30	(70 - 100 - 150)	120 ± 40	(70 - 140 - 150)	110 ± 40	(90 - 110 - 140)
Na (mg/kg)	40 ± 50	(3 - 30 - 200)	8 ± 6	(3 - 5 - 18)	30 ± 10	(10 - 30 - 40)	200 ± 100	(40 - 300 - 500)	100 ± 200	(30 - 40 - 600)	30 ± 20	(10 - 30 - 40)
Mg (mg/kg)	90 ± 40	(10 - 100 - 180)	90 ± 50	(40 - 90 - 160)	100 ± 70	(20 - 90 - 180)	70 ± 30	(30 - 70 - 150)	80 ± 40	(40 - 80 - 150)	80 ± 40	(50 - 80 - 110)
K (mg/kg)	3000 ± 1000	(300 - 3000 - 6000)	3000 ± 1000	(1000 - 3000 - 5000)	3000 ± 2000	(1000 - 3000 - 5000)	2000 ± 1000	(1000 - 2000 - 4000)	2000 ± 1000	(1000 - 2000 - 4000)	3000 ± 1000	(2000 - 3000 - 4000)
Ca (mg/kg)	100 ± 100	(20 - 100 - 800)	30 ± 10	(20 - 30 - 50)	100 ± 40	(60 - 110 - 160)	100 ± 30	(60 - 90 - 130)	90 ± 60	(20 - 110 - 180)	80 ± 10	(70 - 80 - 90)
Al (mg/kg)	7 ± 6	(1 - 5 - 21)	20 ± 20	(1.4 - 10 - 50)	5 ± 3	(3 - 4 - 10)	5 ± 3	(2 - 3 - 13)	9 ± 5	(2 - 10 - 15)	1.7 ± 0.1	(1.7 - 1.7 - 1.7)
Mn (mg/kg)	3 ± 3	(0.5 - 2 - 12)	5 ± 2	(3 - 5 - 7)	10 ± 20	(0.4 - 3 - 30)	3 ± 2	(0.5 - 2 - 7)	1.4 ± 0.6	(0.6 - 1.4 - 2)	2 ± 0.3	(1.9 - 2 - 2.2)
Fe (mg/kg)	4 ± 3	(1 - 4 - 10)	3.1 ± 0.7	(2.3 - 3.2 - 4.5)	4 ± 3	(2 - 3 - 9)	3 ± 2	(1 - 2 - 8)	5 ± 3	(1 - 5 - 9)	1.9 ± 0.3	(1.6 - 1.9 - 2.1)
Ni (mg/kg)	0.3 ± 0.2	(0.05 - 0.3 - 0.7)	0.6 ± 0.7	(0.1 - 0.2 - 2.2)	0.3 ± 0.3	(0.02 - 0.2 - 0.7)	0.09 ± 0.06	(0.05 - 0.07 - 0.28)	0.06 ± 0.01	(0.05 - 0.06 - 0.08)	0.2 ± 0.1	(0.2 - 0.2 - 0.3)
Cu (mg/kg)	1 ± 0.5	(0.1 - 1.1 - 1.9)	1.4 ± 0.5	(0.9 - 1.3 - 2.2)	1.3 ± 0.4	(0.7 - 1.2 - 1.8)	0.6 ± 0.4	(0.2 - 0.5 - 1.8)	0.8 ± 0.3	(0.6 - 0.7 - 1.4)	0.9 ± 0.2	(0.8 - 0.9 - 1.1)
Zn (mg/kg)	1.2 ± 0.6	(0.4 - 1.1 - 2.8)	2 ± 1	(1 - 2 - 4)	1 ± 2	(1 - 1 - 4)	1.1 ± 0.7	(0.7 - 0.9 - 3.3)	1.1 ± 0.5	(0.6 - 1 - 2)	1.09 ± 0.07	(1.04 - 1.09 - 1.13)
Rb (mg/kg)	7 ± 6	(0.04 - 5 - 24)	9 ± 4	(4 - 8 - 14)	4 ± 4	(1 - 2 - 8)	1 ± 1	(0.4 - 1 - 5)	5 ± 3	(1 - 5 - 8)	2.6 ± 0.6	(2.2 - 2.6 - 3)
Sr (mg/kg)	0.3 ± 0.2	(0.04 - 0.2 - 0.7)	0.09 ± 0.04	(0.04 - 0.09 - 0.15)	0.4 ± 0.2	(0.1 - 0.4 - 0.6)	0.24 ± 0.08	(0.13 - 0.23 - 0.4)	0.5 ± 0.2	(0.2 - 0.5 - 0.8)	0.16 ± 0.05	(0.13 - 0.16 - 0.2)
Ba (mg/kg)	0.3 ± 0.2	(0.1 - 0.2 - 0.7)	0.18 ± 0.07	(0.08 - 0.17 - 0.31)	0.5 ± 0.6	(0.1 - 0.2 - 1.7)	0.3 ± 0.3	(0.1 - 0.2 - 0.9)	0.6 ± 0.4	(0.1 - 0.5 - 1)	0.13 ± 0.03	(0.11 - 0.13 - 0.15)
U (ug/kg)	0.5 ± 0.3	(0.1 - 0.4 - 1.1)	0.2 ± 0.2	(0.1 - 0.1 - 0.5)	0.4 ± 0.3	(0.2 - 0.4 - 0.8)	0.3 ± 0.3	(0.1 - 0.2 - 0.7)	0.5 ± 0.4	(0.04 - 0.4 - 1.1)	0.21 ± 0.03	(0.18 - 0.21 - 0.23)
Sc (ug/kg)	1.7 ± 0.8	(0.3 - 1.6 - 3.7)	1.2 ± 0.3	(0.9 - 1.2 - 1.5)	1.4 ± 0.9	(0.4 - 1.2 - 2.7)	1.7 ± 0.8	(0.5 - 1.7 - 2.7)	2 ± 1	(1 - 2 - 4)	1.28 ± 0.08	(1.22 - 1.28 - 1.34)
Y (ug/kg)	5 ± 2	(1 - 4 - 10)	2.7 ± 0.6	(1.8 - 2.5 - 3.4)	4 ± 3	(1 - 3 - 8)	5 ± 3	(1 - 4 - 12)	6 ± 5	(1 - 4 - 14)	2.711 ± 0.004	(2.708 - 2.711 - 2.714)
La (ug/kg)	6 ± 3	(1 - 5 - 12)	2.8 ± 0.5	(2 - 2.7 - 3.6)	4 ± 3	(2 - 3 - 8)	5 ± 3	(1 - 6 - 12)	7 ± 5	(2 - 5 - 15)	2.9 ± 0.4	(2.6 - 2.9 - 3.2)
Ce (ug/kg)	10 ± 8	(1 - 7 - 37)	4 ± 1	(3 - 4 - 6)	6 ± 4	(0.5 - 5 - 12)	9 ± 6	(2 - 8 - 24)	10 ± 9	(3 - 7 - 24)	5.1 ± 0.7	(4.6 - 5.1 - 5.6)
Pr (ug/kg)	1.4 ± 0.7	(0.1 - 1.2 - 2.8)	0.8 ± 0.2	(0.5 - 0.7 - 1)	1.1 ± 0.7	(0.4 - 0.8 - 2.2)	1.4 ± 0.8	(0.4 - 1.6 - 3)	2 ± 1	(0.4 - 1 - 4)	0.8 ± 0.07	(0.75 - 0.8 - 0.85)
Nd (ug/kg)	6 ± 3	(1 - 5 - 12)	3.3 ± 0.8	(2.1 - 3.1 - 4.3)	5 ± 4	(2 - 4 - 9)	6 ± 3	(2 - 7 - 12)	7 ± 6	(2 - 5 - 17)	3.5 ± 0.3	(3.3 - 3.5 - 3.7)
Sm (ug/kg)	1.3 ± 0.6	(0.2 - 1.2 - 2.7)	0.8 ± 0.2	(0.5 - 0.8 - 1.1)	1.1 ± 0.7	(0.4 - 0.8 - 2.1)	1.4 ± 0.7	(0.4 - 1.5 - 2.5)	2 ± 1	(0.4 - 1 - 4)	0.91 ± 0.06	(0.87 - 0.91 - 0.95)
Eu (ug/kg)	1.1 ± 0.5	(0.2 - 1.1 - 2.2)	1 ± 0.3	(0.4 - 1.1 - 1.4)	0.8 ± 0.5	(0.2 - 0.6 - 1.6)	1.1 ± 0.6	(0.3 - 0.9 - 2.3)	0.9 ± 0.5	(0.4 - 0.8 - 1.7)	1.09 ± 0.07	(1.04 - 1.09 - 1.14)
Gd (ug/kg)	1.2 ± 0.5	(0.2 - 1.1 - 2.5)	0.8 ± 0.2	(0.5 - 0.8 - 1)	1 ± 0.7	(0.4 - 0.8 - 2)	1.2 ± 0.6	(0.3 - 1.2 - 2.3)	1 ± 1	(0.4 - 1 - 3)	0.84 ± 0.03	(0.82 - 0.84 - 0.87)
Tb (ug/kg)	0.16 ± 0.08	(0.03 - 0.15 - 0.36)	0.12 ± 0.03	(0.09 - 0.11 - 0.15)	0.1 ± 0.1	(0.1 - 0.1 - 0.3)	0.18 ± 0.09	(0.07 - 0.14 - 0.31)	0.2 ± 0.2	(0.1 - 0.1 - 0.5)	0.12 ± 0.003	(0.117 - 0.12 - 0.122)
Dy (ug/kg)	0.9 ± 0.4	(0.2 - 0.8 - 1.8)	0.6 ± 0.2	(0.4 - 0.6 - 0.8)	0.7 ± 0.5	(0.3 - 0.6 - 1.4)	0.9 ± 0.5	(0.2 - 0.7 - 1.7)	1.1 ± 0.9	(0.3 - 0.7 - 2.6)	0.62 ± 0.04	(0.59 - 0.62 - 0.65)
Ho (ug/kg)	0.14 ± 0.07	(0.02 - 0.12 - 0.31)	0.09 ± 0.02	(0.07 - 0.09 - 0.12)	0.12 ± 0.09	(0.05 - 0.1 - 0.24)	0.15 ± 0.08	(0.05 - 0.12 - 0.3)	0.2 ± 0.2	(0.05 - 0.1 - 0.5)	0.098 ± 0.004	(0.095 - 0.098 - 0.1)
Er (ug/kg)	0.4 ± 0.2	(0.1 - 0.3 - 0.8)	0.23 ± 0.05	(0.16 - 0.22 - 0.29)	0.3 ± 0.3	(0.1 - 0.3 - 0.6)	0.4 ± 0.2	(0.1 - 0.3 - 0.8)	0.5 ± 0.4	(0.1 - 0.3 - 1.2)	0.24 ± 0.01	(0.24 - 0.24 - 0.25)
Tm (ug/kg)	0.06 ± 0.02	(0.01 - 0.07 - 0.1)	0.03 ± 0.01	(0.02 - 0.03 - 0.07)	0.05 ± 0.03	(0.01 - 0.05 - 0.07)	0.06 ± 0.02	(0.02 - 0.06 - 0.09)	0.07 ± 0.06	(0.02 - 0.05 - 0.15)	0.03 ± 0.002	(0.028 - 0.03 - 0.031)
Yb (ug/kg)	0.3 ± 0.1	(0.1 - 0.3 - 0.7)	0.18 ± 0.04	(0.13 - 0.17 - 0.23)	0.3 ± 0.2	(0.1 - 0.2 - 0.5)	0.3 ± 0.2	(0.1 - 0.3 - 0.6)	0.4 ± 0.3	(0.1 - 0.3 - 0.9)	0.2 ± 0.02	(0.18 - 0.2 - 0.21)
Lu (ug/kg)	0.07 ± 0.03	(0.01 - 0.08 - 0.09)	0.03 ± 0.02	(0.02 - 0.08 - 0.08)	0.05 ± 0.04	(0.01 - 0.05 - 0.08)	0.06 ± 0.02	(0.01 - 0.06 - 0.09)	0.07 ± 0.05	(0.01 - 0.06 - 0.14)	0.025 ± 0.003	(0.023 - 0.025 - 0.027)

Table S3. List of the pesticides analyzed

Acetamiprid	DMPF	Tebuconazole
Acetochlor	Ethion	Terbumeton
Achrinatrin	Etofenprox	Terbumeton-desethyl
Alachlor	Fenitrotion	Terbuthylazine
Atrazine	Fenthion	Terbuthylazine-2-hydroxy
Atrazine-desethyl	Fenthion sulfoxide	Terbuthylazine desethyl
Atrazine-deisopropyl	Fenthion sulfone	Tebutryn
Azinphos ethyl	Fipronil	Thiabendazole
Azinphos methyl	Flumethrin	Thiametoxan
Azoxyxtrobin	Fluvalinate	Tolclofos-methyl
Bensulfuron methyl	Hexythiazox	Tricyclazol
Bentazone	Imazalil	
Bifenthrin	Imazamox	
Buprofezin	Imidacloprid	
Carbendazime	Isoproturon	
Carbofuran	Malathion	
Carbofuran-3-hydroxy	MCPA	
Chlorfenvinphos	Methiocarb	
Clorpyrifos	Metoalachlor	
Chlotianidin	Molinate	
Coumaphos	Omethoate	
Cyalofop-buthyl	Parathion ethyl	
Cyhalothrin	Parathion methyl	
Diazinon	Prochloraz	
Dichlofenthion	Propanil	
Difenoconzole	Propazine	
Dimethoate	Pyriproxifen	
Diuron	Simazine	
DMA	Spinetoram	
DMF	Spinosad	
